# Molecular analysis of homeostatic iron regulator, transmembrane protease serine-6, and BTB domain-containing protein-9 variants and iron parameters in blood donors

**DOI:** 10.1042/BSR20202584

**Published:** 2021-01-14

**Authors:** Manal S. Fawzy, Abeer Fakhr-Eldeen, Baraah T. Abu AlSel, Eman A. Toraih

**Affiliations:** 1 Department of Biochemistry, Faculty of Medicine, Northern Border University, Arar, Saudi Arabia; 2 Department of Medical Biochemistry and Molecular Biology, Faculty of Medicine, Suez Canal University, Ismailia, Egypt; 3 Department of Pathology, Faculty of Medicine, Northern Border University, Arar, Saudi Arabia; 4 Department of Clinical Pathology, Faculty of Medicine, Sohag University, Sohag, Egypt; 5 Department of Pathology, Faculty of Medicine, Northern Border University, Arar, Saudi Arabia; 6 Department of Surgery, Tulane University, School of Medicine, New Orleans, Louisiana, U.S.A.; 7 Genetics Unit, Department of Histology and Cell Biology, Faculty of Medicine, Suez Canal University, Ismailia, Egypt

**Keywords:** Allele-specific polymerase chain reaction, blood donors, ferritin, hepcidin, single nucleotide polymorphisms

## Abstract

Genetic variants associated with iron homeostasis have been identified, but their association with iron-related indices and variables among different ethnic populations remains controversial. We aimed to explore the genotype frequency and allelic distribution of three iron-metabolism related variants in homeostatic iron regulator gene (*HFE;* rs1800562 G/A), transmembrane protease, Serine-6 gene (*TMPRSS6*; rs855791 A/G), and BTB domain-containing protein-9 gene (*BTBD9*; rs9357271 C/T) among a sample of the Middle Eastern blood donors and to detect the association of these variants on blood indices, and serum hepcidin/ferritin levels. Real-Time TaqMan genotyping assay for the specified variants was applied for 197 unrelated blood donors. Complete blood picture and serum hepcidin/ferritin levels were assessed. All participants were carriers of rs1800562*G/G genotype for *HFE*. The frequency of A/A and A/G genotypes of *TMPRSS6* rs855791 variant was 55% and 45%, and for C/C, C/T, and T/T of *BTBD9* rs9357271, were 15%, 43%, and 42%, respectively. Minor allele frequencies of rs855791*G and rs9357271*C were 0.23 and 0.37. The GGC genotype combination (for *HFE/TMPRSS6/BTBD9*, respectively) was more frequent in male participants. Higher serum hepcidin and hepcidin/ferritin ratio were observed in *TMPRSS6* (A/G) carriers. While subjects with *BTBD9* C/T and TT genotypes had lower serum ferritin values and higher levels of hepcidin and hepcidin/ferritin ratio compared with C/C genotype. No significant associations were found with any other blood parameters.

In conclusion, *TMPRSS6* rs855791 (A/G) and *BTBD9* rs9357271 (C/T) variants were prevalent in the present blood donor population and may influence the serum hepcidin and/or ferritin levels.

## Introduction

Numerous biologic processes rely upon adequate iron levels [1]. Over the past decade, many traits/disease-associated single-nucleotide polymorphisms (SNPs) have been reported by genome-wide association studies, which have revolutionized our understanding of different genotype–phenotype associations [2].

Data from these studies have identified multiple SNPs associated with changes in serum iron-related parameters and blood cell phenotypes in the general population [3–5]. This influential effect could be explained in part by the intermediate effect on hepcidin and/or ferritin concentrations, although conflicting results in different populations remain [6,7].

Among these SNPs, the *HFE* variants in the human homeostatic iron regulator gene (*HFE*; rs1800562) have been suggested to exert multiple effects on the iron parameters and serum transferrin levels, in part independent of hepcidin [8]. The protein encoded by this gene is an HLA class I-like membrane protein that associates with β2-microglobulin and transferrin receptors on the cell surface [9]. HFE may reduce the affinity of transferrin receptor 1 for transferrin, [10] but its major role is in regulating hepcidin expression, likely via the bone morphogenetic protein (BMP)–SMAD signaling pathway [11].

Another SNP implicated in iron regulation is the transmembrane protease, serine 6 gene (*TMPRSS6*; rs855791), mapped on chromosome 22q12-13, which encodes matriptase-2 (MT-2) [12]. MT-2 is mainly expressed in the liver, where it acts to down-regulate hepcidin expression [13]. It was found to be significantly associated with serum iron, hemoglobin (Hb), the mean corpuscular volume (MCV), and the mean cell hemoglobin (MCH) in genome-wide association studies (GWAS) in some populations [4,14,15].

**Figure 1:**
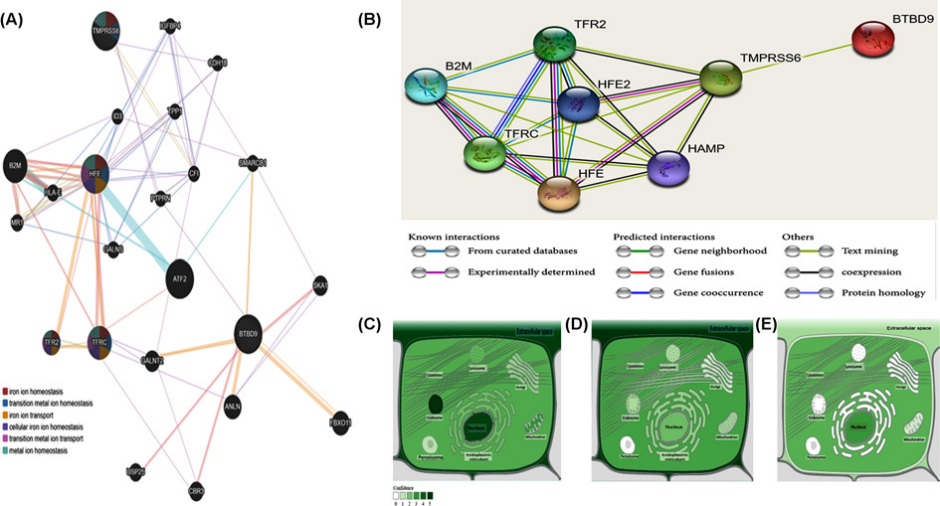
*In silico* data analysis (**A**) Gene–gene functional interaction network generated by GeneMANIA. The network nodes have been colored by function (i.e. gene ontology annotation) (data source: https://genemania.org/search/homo-sapiens/HFE/TMPRSS6/BTBD9). (**B**) Protein–protein interaction using STRING version 11. Each network node represents the protein produced by a single locus. Edges represent protein–protein associations (i.e., contribute to a shared function; this does not necessarily mean they are physically binding to each other). The line colors of the edges indicate the type of interaction evidence that is explained in the figure key. The Network showed a relationship with iron-metabolism related proteins (data source: https://string-db.org). (**C**–**E**) Subcellular localization of HFE, TMPRSS6, and BTBD9 proteins. Darker color, according to the provided color key, is indicating more abundance (data source: https://compartments.jensenlab.org). Abbreviations: ANLN, anillin actin-binding protein; ATF2, activating transcription factor 2; B2M, beta-2-microglobulin; BTBD9, BTB domain containing 9; CBR3, carbonyl reductase 3; CDH18, cadherin 18; CFI, complement factor I; FBXO11, F-box protein 11; GALNS, galactosamine (N-acetyl)-6-sulfatase; GALNT2, polypeptide N-acetylgalactosaminyltransferase 2; HAMP, Hepcidin; HFE, homeostatic iron regulator; ID3, inhibitor of DNA binding 3; IGFBP4, insulin-like growth factor binding protein 4; PTPRN, protein tyrosine phosphatase, receptor type N; SMARCB1, SWI/SNF related, matrix associated, actin-dependent regulator of chromatin, subfamily b, member 1; SKA1, spindle and kinetochore associated complex subunit 1; TFR2, transferrin receptor 2; TFRC, transferrin receptor; TMPRSS6, transmembrane protease, serine 6; TPP1, tripeptidyl peptidase 1; USP25, ubiquitin specific peptidase 25; major histocompatibility complex, class I-related, HLA-E, major histocompatibility complex, class I, E; HLH protein.

Accumulating evidence revealed that some variants related to genes not directly implicated in iron homeostasis might also show an association with iron status parameters. For example, rs9357271 SNP in the BTB domain-containing protein-9 gene (*BTBD9*) was associated with decreased serum ferritin in patients and their relatives; despite the emergence of conflicting results [5,6]. The protein coded by this gene is ubiquitously expressed both in the central nervous system and in the periphery, and both during development and adulthood. [16,17] Although the *BTBD9* variants may directly influence iron metabolism and may be associated with proteins that participate in iron regulation signaling pathways (Figure 1A), yet the mechanism is not known [5].

Based on the earlier works [18,19], serum hepcidin concentration and serum ferritin showed a strong association in both sexes that withstood adjustment for age, BMI, time of blood sampling, and other measured iron parameters like iron and TIBC, as well as biochemical variables such as ALT, eGFR, and CRP [18]. Great interindividual variation in serum hepcidin concentration was observed [20]. This implies that population-based normality values may have limitations when used for the interpretation of hepcidin concentrations. Such it appears that hepcidin values should be interpreted in the context of biochemical tests used to evaluate iron metabolism [21]. Normalization of hepcidin to ferritin levels in the form of hepcidin/ferritin ratio has been evaluated in several genetic studies [22,23]. For example, Dijk et al. found that low serum hepcidin/ferritin ratio was associated with *HFE* C282Y-homozygosity compared with the wild genotype carriers and those with ferritin levels that remain within reference values. These investigators concluded that ‘this ratio might be a useful index for assessing inadequate responses to iron loading and might be of help in predicting which homozygotes may be at risk of developing clinically significant iron loading’ [22]. Additionally, Heeney et al. reported the clinical utility of this ratio in predicting *TMPRSS6* mutation status in patients with chronic iron deficiency [23]. In the case of normal liver function, the hepcidin/ferritin ratio might be a useful indicator of erythropoiesis and iron kinetics, as reported by Lotfi and colleagues [18]. They have realized the importance of this ratio in providing a practical algorithm to predict the spontaneous recovery from iron loss following blood donation [18].

Taken collectively, based on the literature mentioned above and the limited studies that relate the specified SNPs with serum iron-related parameters in the authors’ region, the present study has been conducted to determine the prevalence of rs1800562 in *HFE* (p. Cys282Tyr), rs855791 in *TMPRSS6* (p. Ala736Val) and the intronic variant rs9357271 in *BTBD9* among a sample of Middle Eastern population, and to evaluate the association of these variants with the available iron-related parameters, including the serum hepcidin, ferritin, and hepcidin/ferritin ratio.

The criteria and the selection process of the specified variants are detailed in the ‘Materials and Methods’ section of the present study. The identification and characterization of such associations may lead to better risk stratification for iron-related disorders in this population according to their genetic profile and early implementation of the preventive measurements and individualized iron-based therapeutic strategies for selected one.

## Subjects and methods

### Study participants

The present cross-sectional study enrolled 197 consecutive unrelated blood donors attending the blood bank at the Sohag University Hospital, Sohag, Egypt. The participants selection criteria were age >18 years, non-pregnant, have no history or laboratory findings of chronic disease, inflammatory disorders (leukocyte count more than 10 × 10^3^/μl), history of medication, or any condition could affect or interfere with the measured blood parameters. For each participant, the data related to demographic characteristics, the frequency of blood donation, and the history of iron supplementation have been collected. Donors with missing demographic or genotypic information were excluded. The study has been conducted following the guidelines in the Declaration of Helsinki 2008 and approved by the local Medical and Bioethics Committees of Northern Border University and Sohag University, College of Medicine. Informed consent has been obtained from each participant before taking part.

### Criteria for selecting the study variants and *in silico* data analysis

Literature review and *in silico* approach were applied for retrieving and selection of common (i.e. minor allele frequency > 0.05) SNPs located in genes coding for proteins related to iron-metabolism. Genomic sequence and variants were analyzed in ensemble.org, and the specified variants were selected for each gene (Table 1). Subcellular localization was identified in the Compartments database (compartments.jensenlab.org). Gene–gene interaction was analyzed using Gene Mania version 3.6.0 (https://genemania.org/search/homo-sapiens/HFE/TMPRSS6/BTBD9). Protein–protein association networks and gene ontology were analyzed using String version 11.0 (string-db.org).

**Table 1:** Characteristics of the selected gene variants

Locus	Position	Gene	SNP ID	Alleles	MAF	Type	Citations
6p22.2	6:26092913	*HFE*	rs1800562	G/A	0.06 (A)	missense	222
22q12.3	22:37066896	*TMPRSS6*	rs855791	G/A	0.50 (G)	missense	108
6p21.2	6:38398097	*BTBD9*	rs9357271	T/C	0.50 (T)	intronic	22

Abbreviations: *BTBD9*, BTB domain-containing protein-9 gene; HFE, homeostatic iron regulator gene; MAF, minor allele frequency; SNP, single-nucleotide polymorphism; *TMPRSS6*, transmembrane protease, serine 6 gene. Data are currently mapped to Genome Assembly GRCh38.p13 (Data source: www.ensembl.org)

### Sample collection and the laboratory analysis

Seven milliliters of blood samples were withdrawn from all participants by early day time to minimize the effects of diurnal variation in hepcidin in vacutainer serum separator tubes and ethylenediaminetetraacetic acid (EDTA)-vacutainers. The former tubes were centrifuged at 2500 rpm for 15 min, and the separated sera were subjected to analysis of the routine infectious disease screening according to the local protocols. Ferritin and hepcidin were measured by enzyme-linked Immunosorbent assay Human Ferritin Kit (BioVision, Inc) and Human Hepcidin Quantikine Kit (R& D system) following the manufacturer’s instructions, respectively. The EDTA tubes were used for complete blood count (cell Dyne-3700 fully automated cell counter; Abbott Diagnostics, Wiesbaden, Germany) with blood film examination and for subsequent DNA extraction.

### Iron metabolism-related genes genotyping

Genomic DNA was purified from whole blood using the QIAamp DNA Blood Mini kit (Qiagen, Hilden, Germany) following the manufacturer’s protocol. The concentration and purity of the extracted genomic DNA were assessed by NanoDrop ND-1000 (NanoDrop Technologies, Inc. Wilmington, DE, U.S.A.). Genotyping for the selected three SNPs in iron metabolism-related genes (*HFE; rs1800562; TMPRSS6; rs855791 and BTBD9; rs9357271*) were assayed using TaqMan Real-Time polymerase chain reaction (PCR) allelic discrimination assay as described in details in our previous work [24]. PCR reactions were run in a 25-μl final volume containing 40 ng genomic DNA, TaqMan genotyping Master Mix, and TaqMan SNP Genotyping Assay Mix following the standard protocols. Appropriate controls were used in each reaction plate. The authors who ran the PCR reactions were blinded to the identity of the samples. PCR amplification was done using StepOne™ Real-Time PCR System, and allelic discrimination was called by the SDS software (version 1.3.1, Thermo Fisher Scientific Inc., Waltham, MA, U.S.A.). All quality control measures were applied according to the standard protocols, and replicates of 10% of samples were run with a 100% concordance rate.

### Statistical analysis

Categorical variables were presented as frequency counts and compared using the chi-square test., Meanwhile, the continuous data were expressed as mean ± standard deviation and compared using Student’s *t*-test or Mann–Whitney *U* tests according to data distribution and variance homogeneity. Genotype frequencies for each selected variant were tested for Hardy–Weinberg equilibrium (HWE). The odds ratio (OR) and the 95% confidence interval (CI) were calculated by logistic regression analysis for each variant [25]. Overall and sex-stratified analyses were run. Univariate analysis was performed to test associations. A bivariate correlation matrix using Spearman’s rank correlation analysis was applied to correlate the laboratory results’ different parameters. A multivariate test using the principal component analysis for data exploration was run to test the possibility of participant clustering according to sex and/or genotyping. Statistical significance was set at *P-*value < 0.05. Statistical Package for Social Science software version 23.0 was used for the statistical analysis.

## Results

### Baseline characteristics of the study population

The study enrolled 197 blood donors; 179 men (90.0%) aged 29.1 ± 6.3 years and 18 women (9.1%) aged 30.2 ± 11.6 years old. More than half of the participants (56%) gave blood once for transfusion, 25% donated twice throughout their entire life, while less than a quarter underwent blood donation more than two times. Laboratory results are shown in Supplementary Table S1. No significant differences were found in blood parameters among men and women. In contrast, lower values of MCH and MCHC were observed in young subjects under 30 years old (*P*=0.003 and 0.018, respectively).

### Allelic discrimination analysis

All participants (*n*=197) had rs1800562*G/G genotype for the *HFE* gene. Thus, this variant was not included in the further downstream analysis. On the other hand, rs855791 (A/G) of the *TMPRSS6* gene showed only two genotypes: A/A and A/G in 55% and 45% of the population, respectively, suggesting that this could be due to the small sample size. This later variant was not consistent with HWE in the overall analysis and the male population when stratified by sex (*P*>0.001). Whereas *BTBD9* rs9357271 (C/T) variant was in agreement with HWE in overall and stratified analyses (*P*>0.05) and showed three genotypes: C/C, C/T, as well as T/T accounting for 15%, 43%, and 42% of the sample population, respectively. Minor allele frequencies of rs855791*G and rs9357271*C were 0.23 and 0.37 in the study population (Supplementary Table S2).

On comparing genotype and allele frequencies in men and women, there was no significant difference observed for both *TMPRSS6* rs855791 and *BTBD9* rs9357271 variants (Table 2). Paired loci analysis revealed more frequency of GGC genotype combination in men (Table 3).

**Table 2:** Genetic association models for the study variants

	Genotype	Female	Male	OR (95% CI)	*P-*value
**rs855791**					
Heterozygote comparison	A/A	10 (56)	98 (55)	1.00	0.95
	A/G	8 (44)	81 (45)	1.03 (0.39–2.74)	
Allelic model	A	28 (78)	277 (77)	1.00	0.95
	G	8 (22)	81 (23)	1.02 (0.44–2.33)	
**rs9357271**					
Co-dominant model	T/T	10 (55.6)	72 (40.2)	1.00	0.29
	C/T	7 (38.9)	78 (43.6)	1.55 (0.56–4.28)	
	C/C	1 (5.6)	29 (16.2)	4.03 (0.49–32.90)	
Dominant model	T/T	10 (55.6)	72 (40.2)	1.00	0.21
	C/T-C/C	8 (44.4)	107 (59.8)	1.86 (0.70–4.93)	
Recessive model	T/T-C/T	17 (94.4)	150 (83.8)	1.00	0.18
	C/C	1 (5.6)	29 (16.2)	3.29 (0.42–25.67)	
Over-dominant model	T/T-C/C	11 (61.1)	101 (56.4)	1.00	0.70
	C/T	7 (38.9)	78 (43.6)	1.21 (0.45–3.27)	
Allelic model	T	27 (75)	222 (62)	1.00	
	C	9 (25)	136 (38)	1.83 (0.83–4.02)	0.12
Log-additive	—	—	—	1.77 (0.82–3.82)	0.13

Values are shown as number (percentage). Chi-square or Fisher’s exact tests were used. Abbreviation: OR (95% CI), odds ratio and confidence interval.

**Table 3:** Genotype combination frequencies in men and women

	*HFE*	*TMPRSS6*	*BTBD9*	All	Female	Male	*P*-value	Crude OR (95% CI)	*P*-value
1	G	A	T	0.49	0.52	0.48	0.034	1.00 (reference)	—
2	G	A	C	0.28	0.25	0.29		1.25 (0.64–2.43)	0.499
3	G	G	T	0.14	0.22	0.13		0.64 (0.29–1.41)	0.268
4	G	G	C	0.08	0	0.09		20.56 (1.16–36.29)	**0.039**

Chi-square or Fisher’s exact tests were used. Abbreviations: *BTBD9*, BTB domain-containing protein-9 gene; HFE, homeostatic iron regulator gene; OR (95% CI), odds ratio and confidence interval; *TMPRSS6*, Transmembrane Protease gene, Serine-6. Bold value indicates significance at *P*<0.05.

### Univariate analysis

**Figure 2:**
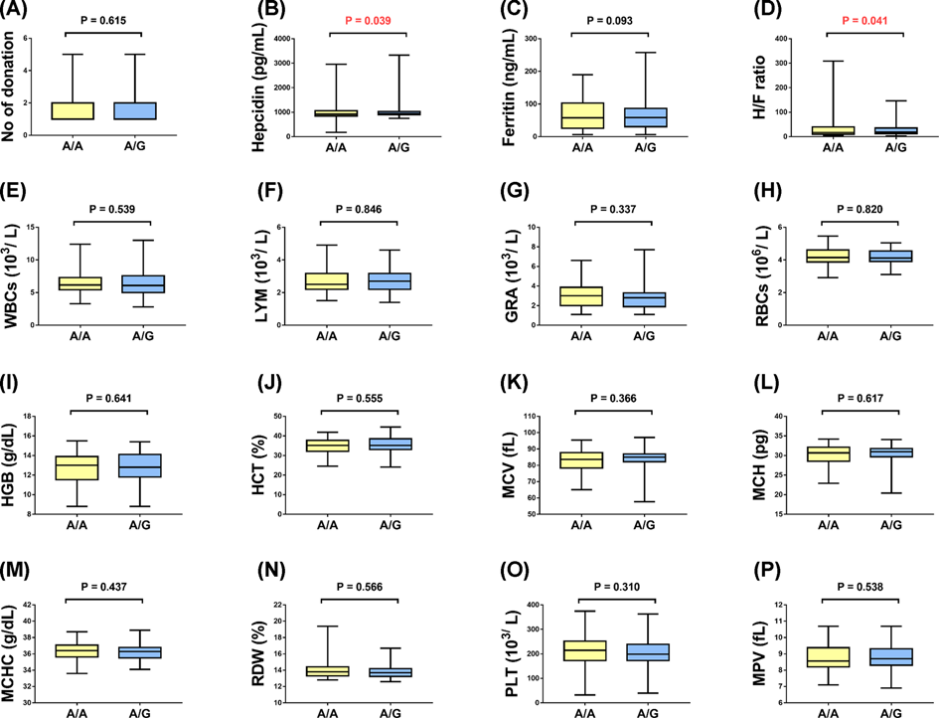
Association between rs855791 *TMPRSS6* and laboratory results (**A**) Number (No) of donation, (**B**) Serum hepcidin (pg/ml), (**C**) Serum ferritin (ng/ml), (**D**) Serum hepcidin/ ferritin ratio, (**E**) White blood cells count (103/L), (**F**) Lymphocyte count (103/L), (**G**) Granulocytes count (103/L), (**H**) Red Blood Cells count (106/L), (**I**) Hemoglobin level (g/dl), (**J**) Hematocrit concentration (%), (**K**) Mean cell volume, (**L**) Mean cell hemoglobin (pg), (**M**) Mean cell hemoglobin concentration (g/dl), (**N**) Red cell distribution width (%), (**O**) Platelets count (103/L), (**P**) Mean Platelet Volume (fL). Box plots represent median and quartile values. Mann–Whitney *U* test was used. *P*<0.05 was set to be statistically significant. Abbreviations: FER, ferritin; GRA, granulocytes; HCT, hematocrit; HEP, hepcidin; H/F, hepcidin/ferritin ratio; HGB, hemoglobin; LYM, lymphocyte; MCH, mean cell hemoglobin; MCHC, mean cell hemoglobin concentration; MCV, mean red cell volume; MPV, mean platelet volume; PLT, platelets; RBC, red blood cells; RDW, red cell distribution width; WBC, white blood cells.

**Figure 3:**
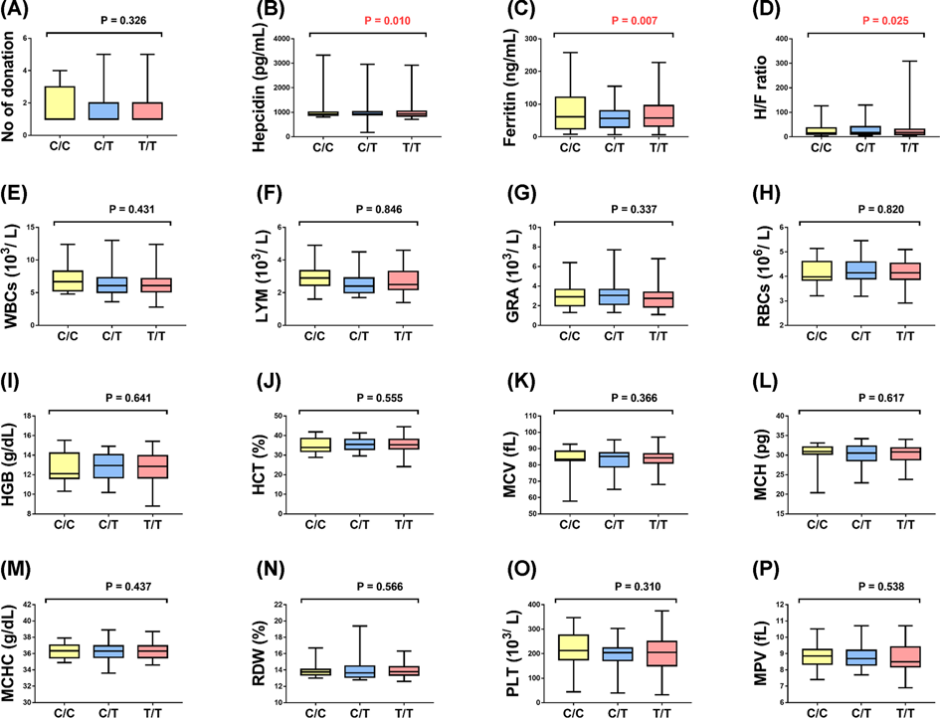
Association between rs9357271 *BTBD9* and laboratory results (**A**) Number (No) of donation, (**B**) Serum hepcidin (pg/ml), (**C**) Serum ferritin (ng/ml), (**D**) Serum hepcidin/ ferritin ratio, (**E**) White blood cells count (103/L), (**F**) Lymphocyte count (103/L), (**G**) Granulocytes count (103/L), (**H**) Red Blood Cells count (106/L), (**I**) Hemoglobin level (g/dl), (**J**) Hematocrit concentration (%), (**K**) Mean cell volume, (**L**) Mean cell hemoglobin (pg), (**M**) Mean cell hemoglobin concentration (g/dl), (**N**) Red cell distribution width (%), (**O**) Platelets count (103/L), (**P**) Mean Platelet Volume (fL). Box plots represent median and quartile values. Mann–Whitney U test was used. *P*<0.05 was set to be statistically significant. Abbreviations: FER, ferritin; GRA, granulocytes; HCT, hematocrit; HEP, hepcidin; H/F, hepcidin/ferritin ratio; HGB, hemoglobin; LYM, lymphocyte; MCH, mean cell hemoglobin; MCHC, mean cell hemoglobin concentration; MCV, mean red cell volume; MPV, mean platelet volume; PLT, platelets; RBC, red blood cells; RDW, red cell distribution width; WBC, white blood cells.

Associations of rs855791 *TMPRSS6* and rs9357271 *BTBD9* variants with biochemical results were depicted in Figure 2 and Figure 3. For *TMPRSS6* gene, A/G genotype carriers exhibited higher levels of serum hepcidin (*P*=0.039) and hepcidin/ferritin ratio (*P*=0.041). While in *BTBD9* gene, subjects with C/T and TT genotypes had lower values of serum ferritin (*P*=0.007) and higher levels of hepcidin (*P*=0.010) and hepcidin/ferritin ratio (*P*=0.025) compared with C/C genotype. No significant associations were found with any other blood parameters.

### Multivariate analysis

**Figure 4:**
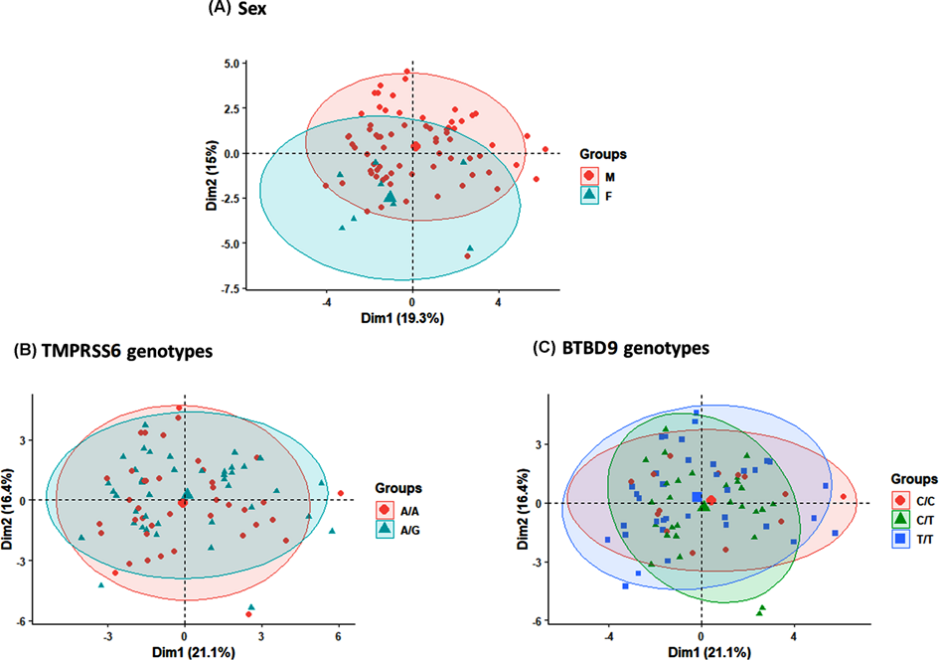
Principal component analysis No clear demarcation was found between males and females (**A**) or subjects with different genotypes (**B** and** C**).

As illustrated in Figure 4, the principal component analysis did not show discrete clustering of individuals based on their sex or genotypes.

## Discussion

There is a growing body of literature that recognizes the implication of multiple SNPs in several aspects of iron metabolism through known and yet unknown mechanisms or to be associated with some iron-related regulatory pathways [26]. In this sense, we explored in the present study the association of three iron homeostasis-related SNPs on the iron parameters and serum hepcidin/ferritin levels in a cohort of Middle Eastern donors. We show that the SNPs *TMPRSS6*; rs855791 and *BTBD9*; rs9357271 were potentially associated with variations in serum hepcidin, serum ferritin, and/or hepcidin normalized to ferritin (the hepcidin/ferritin ratio).

Although some studies, including the one done by Traglia et al. [8] and replicated by Galesloot et al. [4] in the Italian population, postulated the implication of *HFE*; rs1800562 in several aspects of iron parameters through hepcidin-dependent and independent mechanisms. Currently, this specified SNP in *HFE* gene could not be further analyzed because of its monomorphic genotype (GG; 100%) in the present population. However, this finding was in line with previous studies that failed to detect this variant in adult hepatitis C virus-induced liver cirrhosis, hemochromatosis, pediatric lymphoblastic leukemia survivors, or the controls in the same region [27–29]. These studies, including ours, are consistent with that rs1800562 (G/A) frequency, in general, ranged from 0 to 9.9% and seen to be nearly 0% in the North African population. Further large-scale multicenter studies are warranted to confirm this finding.

Several studies have realized the associations of the *TMPRSS6* variants with decreased serum iron, [14] serum ferritin, [30], and Hb levels [14,31,32]. The current study showed that A/G genotype carriers of this variant have higher levels of serum hepcidin and hepcidin/ferritin ratio compared with the wild genotype carriers. Although this variant showed a departure from HWE, authors did not reject this SNP from further analysis as they speculated this could be attributed to the missing of the homozygous genotype of this SNP minor allele in the study population, according to Lewis and Knight affirmative conclusion that ‘No standard guidelines for rejecting SNPs that depart from HWE have been developed’ [33].

Given the significant role *TMPRSS6* plays in iron regulation by coding for the hepatic serine protease (MTP-2) that can modulate the serum hepcidin levels in normal individuals [34] and regulate the hepatic production of hepcidin, [35] this could explain the present association of the study missense rs855791variant with the serum hepcidin and hepcidin/ferritin ratio. Consistent with others, Nai et al. [34] found that the C genotype of this specified variant could inhibit hepcidin more efficiently than the T genotype in their in *vitro* experiment, and the CC homozygous carriers had lower serum hepcidin levels and higher transferrin saturation than those with TT homozygotes in the general population [34].

Regards *BTBD9* C/T variant, associated with periodic limb movements in sleep (restless leg syndrome), currently individuals with C/T and TT genotypes have lower values of serum ferritin and higher levels of hepcidin and hepcidin/ferritin ratio compared with C/C genotype. Although the function of BTBD9 protein was not described, and its exact role in the context of iron homeostasis remains to be elucidated [24]. Our finding was in line with the previous studies that reported an association of this variant with serum ferritin level in subjects with restless leg syndrome and their relatives [6,36]. Serum ferritin levels could decrease by nearly 13% parallel to the at-risk SNP in this specified gene [36]. However, it is worth noting that even where statistically significant differences in laboratory parameters were observed between different genotypes, the differences were so small that their physiological significance is questionable.

Our multivariate analysis could not discriminate or cluster the study population based on their genotype. Excluding the relatively small sample size, this may reflect that simultaneous testing multiple variants (not just three variants) may be optimal for determining the genetic background's contribution to different phenotypes, at least in some populations. We need in the future to run such type of large-scale genomic analysis, which can lead to ‘discoveries that may be hidden in individual analyses of a single or few sources’ as proposed recently by Allen [37].

In conclusion, the present study demonstrated that *TMPRSS6* rs855791 (A/G) and *BTBD9* rs9357271 (C/T) variants were associated with serum hepcidin and/or serum ferritin levels in a preliminary sample of Middle Eastern blood donors. However, the present study could be limited by the relatively small sample size and the study design’s cross-sectional analysis. Large-scale, multi-central, and prospective follow-up studies are warranted to verify the current conclusions.

## Supplementary Material

Supplementary Tables S1-S2

## Data Availability

All data used were included in the manuscript, and additional results were available in the Supplementary Material.

## Abbreviations

BMP: bone morphogenetic protein
*BTBD9*: BTB domain-containing protein-9 gene
CI: confidence interval
GWAS: genome-wide association studies
Hb: hemoglobin
HWE: Hardy–Weinberg equilibrium
MCH: mean cell hemoglobin
MCV: mean corpuscular volume
MT-2: matriptase-2
OR: odds ratio
SNP: single-nucleotide polymorphism
