# Supplementary material for: Molecular analysis of homeostatic iron regulator, transmembrane protease serine-6, and BTB domain-containing protein-9 variants and iron parameters in blood donors
Source: Biosci Rep. 2021 Jan 14;41(1):BSR20202584. doi: 10.1042/BSR20202584 (PMC7809544; doi:10.1042/BSR20202584)
Supplement: Supplementary Tables S1-S2 [file BSR-2020-2584_supp.pdf]

Table S1. Hematological results of the study participant

	Overall		Distribution		Sex					Age*					Number of donations				
	Total (n=197)		Skew	Kur	Women (n=18)		Men (n=179)		*P*-value	<30 y (n=104)		≥30 y (n=93)		*P*-value	Once (n=112)		≥2 times (n=88)		*P*-value
	**Mean**	**SD**			**Mean**	**SD**	**Mean**	**SD**		**Mean**	**SD**	**Mean**	**SD**		**Mean**	**SD**	**Mean**	**SD**	
HEP pg/mL	539	623	1.28	2.76	541	870	539	596	0.693	580.6	645	493	598	0.264	936	483	1021.7	625.8	0.781
FER ng/mL	33.4	47.6	1.81	3.78	31.9	46.2	33.5	47.9	0.629	29.4	37.9	37.9	56.4	0.968	61.6	49.2	58.1	54.4	0.500
H/F	15.6	32.1	5.14	37.7	7.2	10.5	16.4	33.5	0.269	20.2	40.9	10.4	16.7	0.097	23.7	28.0	35.9	51.8	0.171
WBC	6.5	1.9	1.11	1.79	5.5	2.5	6.6	1.9	0.152	6.4	1.5	6.7	2.4	0.363	6.8	2.0	6.3	2.0	0.274
LYM	2.7	0.7	0.76	0.34	2.6	0.6	2.7	0.7	0.567	2.7	0.7	2.8	0.8	0.537	2.6	0.7	2.8	0.8	0.173
MID	0.8	0.3	1.31	2.33	0.6	0.3	0.8	0.3	0.189	0.7	0.2	0.9	0.4	0.232	0.8	0.3	0.8	0.3	0.261
GRA	3.0	1.4	1.19	1.58	2.3	2.0	3.1	1.3	0.172	3.0	1.1	3.1	1.6	0.588	3.3	1.4	2.7	1.4	0.035
LYM%	42.5	10.5	-0.29	0.40	49.8	12.4	42.0	10.2	0.056	42.0	10.4	43.2	10.7	0.588	39.2	11.1	46.2	8.6	0.001
MID%	12.0	3.1	1.33	2.28	11.8	2.4	12.0	3.2	0.957	11.6	2.7	12.5	3.6	0.378	12.2	3.4	12.1	2.9	0.817
GRA%	45.1	9.8	0.02	-0.86	38.4	12.4	45.6	9.4	0.061	45.6	9.9	44.4	9.6	0.526	47.7	9.5	41.7	9.2	0.003
RBC	4.2	0.5	-0.02	0.10	4.1	0.3	4.2	0.5	0.530	4.2	0.4	4.2	0.6	0.643	4.3	0.5	4.1	0.5	0.182
HB g/dL	12.7	1.5	-0.45	-0.28	12.6	1.1	12.7	1.5	0.884	12.5	1.4	13.0	1.6	0.131	13.0	1.4	12.4	1.6	0.071
HCT	35.1	3.9	-0.32	0.15	34.7	2.5	35.1	4.0	0.769	34.7	3.7	35.5	4.2	0.306	35.6	3.6	34.1	4.2	0.076
MCV	83.7	6.2	-1.11	3.00	84.8	4.0	83.7	6.4	0.656	83.1	5.3	84.5	7.2	0.142	83.9	6.0	83.1	6.8	0.618
MCH	30.4	2.4	-1.38	2.92	30.9	1.7	30.3	2.5	0.685	30.0	2.1	30.9	2.7	0.003	30.5	2.3	30.2	2.7	0.531
MCHC	36.3	1.0	0.07	-0.14	36.4	0.8	36.2	1.0	0.615	36.0	0.9	36.5	1.1	0.018	36.4	1.0	36.3	1.1	0.639
RDW	13.9	1.0	2.31	8.73	13.5	0.4	14.0	1.0	0.176	13.9	0.9	14.0	1.1	0.694	14.0	1.1	13.9	0.9	0.525
PLT	205	68.9	-0.15	0.41	244.4	97.1	202.9	66.1	0.125	210.4	75.7	200.0	59.6	0.457	207.0	70.2	204.0	70.9	0.843
MPV	8.8	0.7	0.38	0.19	8.4	0.8	8.8	0.7	0.197	8.7	0.6	8.9	0.9	0.242	8.6	0.7	9.0	0.8	0.015

Data are represented as mean and standard deviation (SD). Skewness (skew) and kurtosis (Kur) coefficients are considered acceptable between -2 and +2. In this case, a student t-test was applied. Otherwise, the Mann-Whitney U test was used.*The age of 30 years cut off was selected based on the mean age of the study participants. $P<0.05$ was set to be statistically significant.HEP: hepcidin, FER: ferritin, H/F: hepcidin/ferritin ratio, WBC: White Blood Cells, LYM: lymphocyte, GRA: granulocytes, RBC: Red Blood Cells, HGB: Hemoglobin, HCT: Hematocrit, MCV: Mean Red Cell Volume, MCH: Mean Cell Hemoglobin, MCHC: Mean Cell Hemoglobin Concentration, RDW: Red Cell Distribution Width, PLT: Platelets, and MPV: Mean Platelet Volume.

TABLE S2 Genotype and allele frequencies of Transmembrane Protease, Serine 6 (*TMPRSS6*),and BTB domain-containing protein gene (*BTBD*) stratified by sex

	Genotype				Alleles		
**rs855791**	**A/A**	**A/G**	**G/G**	***P*-value**	**A**	**G**	***P*-value**
All	108 (55)	89 (45)	0 (0.0)	---	305 (77)	89 (23)	---
Female	10 (56)	8 (44)	0 (0.0)	0.947	28 (78)	8 (22)	0.956
Male	98 (55)	81 (45)	0 (0.0)		277 (77)	81 (23)	---
**rs9357271**	**C/C**	**C/T**	**T/T**	***P*-value**	**C**	**T**	***P*-value**
All	30 (15)	85 (43)	82 (42)	---	145 (37)	249 (63)	---
Female	1 (6)	7 (39)	10 (56)	0.328	9 (25)	27 (75)	0.123
Male	29 (16)	78 (44)	72 (40)		136 (38)	222 (62)	---

Data are shown as number (percentage). A Chi-square test was used for analysis.
